# Effectiveness of a novel mobile health education intervention (Peek) on spectacle wear among children in India: study protocol for a randomized controlled trial

**DOI:** 10.1186/s13063-017-1888-5

**Published:** 2017-04-08

**Authors:** Priya Morjaria, Andrew Bastawrous, Gudlavalleti Venkata Satyanarayana Murthy, Jennifer Evans, Clare Gilbert

**Affiliations:** 1 Faculty of Infectious and Tropical Diseases, Department of Clinical Research, London School of Hygiene and Tropical Medicine, Keppel Street, London, WC1E 7HT UK; 2 Indian Institute of Public Health, Plot No #1, A.N.V. Arcade, Amar Co-op Society, Kavuri Hills, Madhapur, Hyderabad, 500033 India

**Keywords:** Uncorrected refractive errors, Children, School eye health, India, Spectacle wearing rate, Health education, Portable eye examination kit, Peek, Randomized clinical trial

## Abstract

**Background:**

Uncorrected refractive errors are the commonest cause of visual loss in children despite spectacle correction being highly cost-effective. Many affected children do not benefit from correction as a high proportion do not wear their spectacles. Reasons for non-wear include parental attitudes, overprescribing and children being teased/bullied. Most school programmes do not provide health education for affected children, their peers, teachers or parents.

The Portable Eye Examination Kit (Peek) will be used in this study. Peek has applications for measuring visual acuity with software for data entry and sending automated messages to inform providers and parents. Peek also has an application which simulates the visual blur of uncorrected refractive error (SightSim).

The hypothesis is that higher proportion of children with uncorrected refractive errors in schools allocated to the Peek educational package will wear their spectacles 3–4 months after they are dispensed, and a higher proportion of children identified with other eye conditions will access services, compared with schools receiving standard school screening.

**Methods/Design:**

Cluster randomized, double-masked trial of children with and without uncorrected refractive errors or other eye conditions. Government schools in Hyderabad, India will be allocated to intervention (Peek) or comparator (standard programme) arms before vision screening. In the intervention arm Peek will be used for vision screening, SightSim images will be used in classroom teaching and will be taken home by children, and voice messages will be sent to parents of children requiring spectacles or referral.

In both arms the same criteria for recruitment, prescribing and dispensing spectacles will be used. After 3–4 months children dispensed spectacles will be followed up to assess spectacle wear, and uptake of referrals will be ascertained.

The cost of developing and delivering the Peek package will be assessed. The cost per child wearing their spectacles or accessing services will be compared.

**Discussion:**

Educating parents, teachers and children about refractive errors and the importance of wearing spectacles has the potential to increase spectacle wear amongst children. Innovative, potentially scalable mobile technology (Peek) will be used to screen, provide health education, track spectacle wear and adherence to follow-up amongst children referred.

**Trial registration:**

Controlled-Trials.com, ISRCTN78134921. Registered on 29 June 2016.

**Electronic supplementary material:**

The online version of this article (doi:10.1186/s13063-017-1888-5) contains supplementary material, which is available to authorized users.

## Background

Uncorrected refractive errors (uRE) are the commonest cause of visual loss in children.

The proportion of visual impairment due to uREs, defined as ≤6/12 in the better eye, in a group of standardized studies of children aged 5 to 15 years was 56.0% in Nepal [1], 56.3% in Chile [2], 61.0% in rural India [3], 63.6% in South Africa [4], 81.7% in urban India [5], 87.0% in Malaysia [6], 89.5% in rural China [7] and 94.9% in urban China [8]. Correcting refractive errors (RE) is highly cost-effective [9–11]. It is estimated that 12.8 million children worldwide are visually impaired from uRE [12].

Refractive errors can result from the axial length of the eye being too long or too short, and from abnormalities in the curvature of the cornea. The three most common types of REs are myopia (short-sightedness), hypermetropia (long-sightedness) and astigmatism (distorted vision at distance and near). Myopia is the commonest type of RE [13]. The onset is usually around the age of 8 years and increases in severity throughout adolescence [13]. Myopia is far more common in Southeast Asian children, where the age of onset is earlier and progression more rapid [13]. There is increasing evidence of the impact of correcting RE in children, with improvement in social development, quality of life, visual functioning and academic performance [14].

In India, there are approximately 140 million children aged 11–15 years, 5.6 million (4%) of whom have uRE and would benefit from spectacles. Correction of REs is a priority of the Government of India [15]. However, a high proportion of children who could benefit from RE correction do not wear their spectacles [16], reported as only 30% in a recent study in India [17]. There are many reasons for non-wear, including parents not purchasing spectacles, overprescribing and children being teased and bullied or not liking their spectacles [16]. Some parents fear that spectacles will weaken their child’s eyes, are expensive and are stigmatizing, or indicate that their child has a disability [18]. In India, some programmes have trained teachers to screen vision, but teachers are not usually otherwise engaged in the process and they usually do not promote or monitor spectacle wear. It is not standard practice in India to send explanatory pamphlets to parents of children requiring spectacles and parents are not typically made aware of the benefits of spectacle wear.

There have been three trials of interventions to improve spectacle wear: an education intervention of students in China, which had negative results, showing that educating children alone is not effective [19]. Another recent trial in China had a factorial design with six subgroups. Children in half the schools were randomized to a health education intervention, which involved showing children a 10-minute documentary-style video, a booklet of cartoons, and classroom discussion led by teachers. The same schools were randomized to three approaches to providing spectacles i.e. free spectacles, a voucher, or children were given a prescription for spectacles. Spectacle wear was assessed by observation and self-report. Observed wear was higher in all four subgroups randomized to the health education intervention (RR 1.46 to 1.74) [14]. The other trial was of free versus low-cost spectacles in Tanzania, in which free spectacles almost doubled wearing rates [18].

A recent trial by the investigators in Bangalore, India showed that 2.6% of children aged 11–15 years had significant uRE, defined as a level of visual loss due to RE which improved by two or more lines of Snellen visual acuity (VA) in one or both eyes with spectacle correction. In this study, children could select the spectacle frames they preferred and almost 75% were wearing their spectacles at unannounced visits 3–4 months later [20].

Mobile phone technology is a rapidly expanding area in health care [21], including eye care [22]. A recent development, the Portable Eye Examination Kit (Peek) [23] has a suite of applications (apps), including for measuring VA [24] which has been found to be an acceptable tool for patients, examiners and stakeholders in a recent study in Kenya [25]. The Peek School Screening system enables automated text and voice messaging to parents/guardians and contact teachers as well as real-time notifications to refractive or hospital services of screened positive children who require further assessment or follow-up. Peek also has an app which generates images that simulate the visual blur associated with uRE (SightSim). (See Additional file 1: Figure S1.) A recent trial in schools in Kenya using the system demonstrated that teachers could be taught to screen VA reliably using the Peek app, and SightSim images (Polaroid photographs) and text messages were sent to parents. Uptake of referrals to eye care providers was two and a half times higher in the Peek intervention arm of the trial (unpublished data).

Research on why children with significant refractive errors do not wear their spectacles is limited, but the available evidence highlights the importance of environmental factors, particularly the negative attitudes of others i.e., peers, parents, the wider family and teachers, as well as community norms and attitudes [26, 27]. There are multiple theories and constructs which can be used to describe behavior or to bring about behavior change, and the Social Ecological Model (SEM), which describes five nested, hierarchical levels which influence behavior i.e., individual, interpersonal, organizational, community and public policy, has been adopted in this study as it encapsulates the main factors which influence spectacle wear among children [28]. The SEM emphasizes that it is easier to adopt healthier behaviors by bringing about change in the environment, by using the example of role models, and by reinforcement. In the trial being planned, SightSim images of relevance to Indian children aged 11–15 years will be used, including images of role models such as sports personalities, and used in classroom teaching so that all children as well as teachers learn about the impact of uRE and the impact of correction. Children identified who need spectacles will also take home a SightSim image of their choice to show their parents, which demonstrates how much clearer their child’s world would be if they wore their spectacles. Messages will be reinforced to parents through voice messages send to their mobile phones.

### Purpose

The purpose of this cluster randomized trial is to evaluate whether a health education package for teachers, parents and children (aged 11–15 years), delivered using Peek increases spectacle wear at 3 to 4 months and uptake of referral of children identified with other eye conditions.

The trial will also assess the cost of developing and delivering the Peek health education intervention and the cost of dispensing and delivering the spectacles in both arms of the trial. The cost per child wearing their spectacles at follow-up will be compared between the two arms of the trial.

The hypothesis is that the proportion of children wearing spectacles 3–4 months after they were given their spectacles is higher in schools allocated to the innovative Peek educational package than in schools randomized to the standard programme. The uptake of referrals is also anticipated to be higher in the schools allocated to the Peek educational package than those randomized to the standard programme.

### Formative research and pilot study November 2016

Formative research including a pilot study will be undertaken in non-trial schools to test all aspects of the trial, including which SightSim images to use and the content of voice messages to parents to remind them to encourage their children to wear their spectacles, or to access services. The formative research will use mixed methods, including focus group discussions separately with head teachers, parents of both boys and girls of different ages and also boys and girls of different ages. Focus group discussions with head teachers will gather their views of spectacle wear by children, views on using SightSim images to increase awareness among parents, and which images they consider the most suitable for parents.

Based on data from the focus group discussions, images will be selected for classroom teaching. These classroom education materials will be shown to children and they will be asked to give their opinion on activities that children with uncorrected refractive error might like to do but cannot do because they do not have clear enough vision. Children will be shown SightSim images of the visual blur experienced by children with uRE and they will be asked to express their reactions. Classroom teachers will also be asked to comment on the images suggested.

A short questionnaire to assess children’s knowledge of and attitudes towards spectacle wear will be assessed immediately before and after a session of classroom teaching using SightSim images. In each school, two classes of different ages will be administered the questions before and after classroom teaching, a total of approximately 200 children aged 11–15 years.

## Methods/design

The trial is designed as a cluster randomized, double-masked clinical trial of children with and without uRE in accordance with the Standard Protocol Items: Recommendations for Interventional Trials (SPIRIT) guidelines [29]. Children will be masked to the allocation as they will not be told that other schools will have a different intervention, and field workers who collect the data for the primary outcome will also be masked to the hypothesis and intervention arm.

### Study setting

The trial is being undertaken in government middle and secondary schools in urban and rural areas in and around Hyderabad, Telangana State, India. The trial is being coordinated by the Public Health Foundation of India (PHFI), Hyderabad. The team consists of a programme manager, administrator, optometrists, dispensing opticians, field workers and ophthalmologists. Training, quality assurance and oversight of data collection are being provided by staff at the International Centre for Eye Health, London School of Hygiene & Tropical Medicine (LSHTM).

### Participant eligibility

#### Selection of schools

A list of government secondary schools will be obtained from the District Block Education Officer. The precise location of each school will be determined using Google Maps. Schools will be excluded if they already have school eye health programmes where screening took place within the previous 2 years, or are single-gender schools. If two schools are less than 10 km apart one will be excluded at random. Schools will be stratified by location (urban/rural) and size (more or less than 200 children aged 11–15 years).

The head teacher of each selected school will be visited by a field worker to obtain written informed consent for the school to participate. An information sheet in the local language with an opt-out option will be given to each child aged 11–15 years for them to take home. Parents will be given the option to opt out entirely i.e., that their child is not screened, or to opt out from being recruited to the trial.

#### Inclusion criteria

There are two mutually exclusive eligibility criteria in this study i.e. for spectacle correction and for referral. Common criteria for both are that children are aged 11–15 years inclusive, parents’ consent for their child to take part in the study and the child proves assent, and they have a presenting VA (i.e. with spectacles if usually worn) of less than 6/9.5 in one or both eyes. Children will be eligible for immediate spectacle correction if their binocular VA with full correction improves by two or more lines. Children will be eligible for referral if they require cycloplegic refraction, if their presenting VA is 6/60 or less in one or both eyes regardless of the cause, if their best-corrected visual acuity does not improve by two or more lines in both eyes or they require further investigation for any other non-refractive eye conditions.

#### Exclusion criteria

Children will not be recruited if parents do not consent or the child does not assent. Children whose parents agree that they can be screened will be assessed and spectacles dispensed if required, or they will be referred, but will not be recruited to the trial. Children whose parents opt out entirely, but where the teacher suspects a problem will be given a letter to take home.

### Eligibility of those performing interventions

All refractions, prescribing and dispensing are being undertaken by fully qualified optometrists, with training and quality checks by the lead investigator. All screening, delivery of spectacles and follow-up at 3–4 months will be conducted by trained field workers.

### Identification of potential participants and recruitment

In the intervention arm, field investigators in the school identified by the head teacher will first undertake classroom teaching using the SightSim images for all children aged 11–15 years, after training. A training manual will be developed to standardize the delivery. Field workers will be trained in each school to screen VA using the Peek vision screener app at the Snellen equivalent 6/9.5 level of VA, and children aged 11–15 years will be screened. To pass vision screening a child needs to correctly see four or five of five consecutive E optotypes. Once screening has been completed, all those who fail will be automatically referred to a visiting optometrist and will be refracted on the same day. Attendance will be checked against the data in the Peek software. After identifying children requiring spectacles each child will select a SightSim image of their choice from a range of images, which they will be asked to take it home to show their parents.

**Fig. 1:**
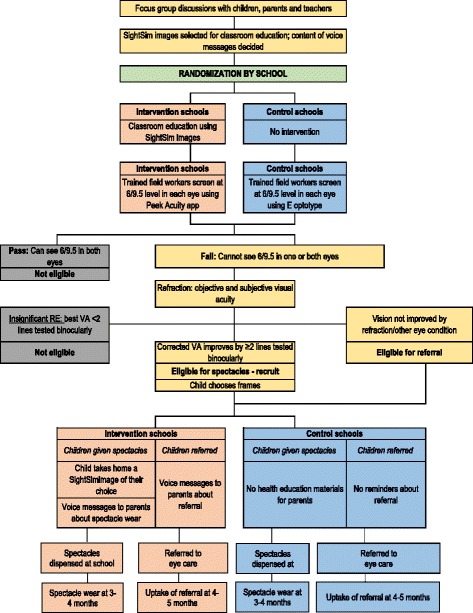
Study flowchart

In the schools allocated to the comparator arm, trained field workers will screen VA using a standard card-based E optotype at the 6/9.5 level at 6 meters. To pass at the 6/9.5 level children need to correctly indicate the orientation of the E in at least four out of five optotypes. No health education materials or voice messages will be sent to the parents (Fig. 1).

To standardize data recording, data will be entered using dedicated Peek software in both arms of the trial. The software will have range and consistency checks built in. Field workers and optometrists working in schools in both arms of the trial will be given tablets for data entry, and data will be regularly backed up onto a cloud server. In both arms of the trial the head teacher will be given a list of all the children in their school who require spectacles or referral. Classroom teachers will also be given a list of children in their class who require spectacles or referral.

The following information will be collected by trained field workers by interviewing all eligible children in both arms of the trial: name, age, gender, class and date of birth; parents’ mobile phone number, language used in the home; parental education; occupation of parents; whether one or both parents wear spectacles for distance vision and limited information on household assets taken from National Household Survey questionnaires. Data on subjective refraction and best-corrected VA in each eye will be recorded as well as the prescription of the spectacles required and the frame the child selected from a selection of thirteen plastic frames of different colours. In the intervention arm, the SightSim image the child selects to take home will be recorded.

In both groups of schools, only children with significant REs will be prescribed spectacles i.e., where after correction, the acuity improves by two or more lines binocularly. Children will select the frames they prefer from a range of coloured plastic frames. Each pair of spectacles will have a unique code. Spectacles will be delivered to the schools by field workers at no cost as soon as possible and all children in each school will receive their spectacles on the same day. At the school, field workers will double-check the name of each child against the lists given to classroom teachers, to ensure that each child receives the correct spectacles. The field workers will measure children’s VA with their new spectacles.

In both arms of the trial, children with other eye conditions will be referred using a referral slip indicating their study ID, name and school, indicating that assessment and treatment will be provided at no cost at Pushpagiri Eye Institute on presentation of the referral slip. An administrator will be appointed at Pushpagiri Eye Institute who will access lists generated by Peek software of all children referred. The administrator will enter the date of attendance of children who attend Pushpagiri Eye Institute into Peek software for children referred from the intervention and comparator arms of the trial.

In the intervention arm of the trial, voice messages will be sent to parents of children requiring spectacles in the local languages within 1 week of their child being given their spectacles and again every 2 weeks for 3 months. Reminder messages will also be sent to parents of children referred within 1 week of referral and again every 2 weeks for 3 months if their child has not attended.

Fieldwork has been planned such that the initial assessment, delivery of spectacles and follow-up 3–4 months later do not coincide with school examination periods, long school holidays, nor the end of the school year when children may leave or change schools.

If the Peek education package is found to be superior to the control schools, the same package will be delivered after the 3–4 month follow-up.

### Participant timeline and study flowchart

**Fig. 2:**
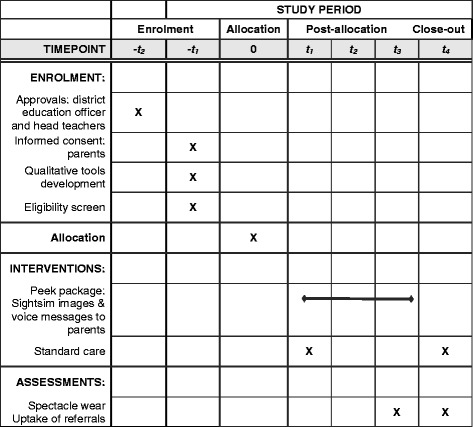
SPIRIT timeline

The study flowchart and participant timeline are presented in Figs. 1 and 2, respectively. A filled Standard Protocol Items: Recommendations for Interventional Trials (SPIRIT) checklist is available (see Additional file 2).

### Intervention and comparator arm

The intervention will be the Peek Acuity health education package. The comparator will be a standard school screening programme where no health education materials or messages are sent to parents (standard care) (Table 1).

**Table 1:** An overview of the two arms of the trial

	Intervention arm	Comparator arm
Age group	11–15 years	11–15 years
Screening VA level	<6/9.5 in one or both eyes	<6/9.5 in one or both eyes
Method of screening	Peek E Acuity by field workers	E card optotype by field workers
Health education	• SightSim images for classroom teaching by field workers, after orientation i.e. for all children	None
	• Eligible children will select a SightSim image of their choice from a range of pre-tested images to take home to show their parents with wording in the relevant local language	
	• Personalized voice messages for parents in the relevant local language	
Refraction	Trained optometrist	Trained optometrist
Definition of significant RE	Same in both arms of the trial	Same in both arms of the trial
Dispensing criteria	Same in both arms of the trial	Same in both arms of the trial
Frame types	Same in both arms of the trial	Same in both arms of the trial
Delivery of spectacles	Same in both arms of the trial	Same in both arms of the trial
Assessment of primary outcome	Same in both arms of the trial	Same in both arms of the trial

*VA* visual acuity, *Peek* Portable Eye Examination Kit, *RE* refractive errors

### Sample size calculation

A superiority margin of 20% was chosen to balance the anticipated higher costs of developing and delivering the Peek package. We estimate that we will need a study size of 450 children (225 in each arm) to detect a difference of 20% in spectacle wear between the intervention and comparator arm, assuming approximately 60% of children in the control arm will be wearing spectacles at follow-up, with a 95% confidence interval and 90% power. We have adjusted the sample size calculations for clustering using data from our previous study (unpublished) to estimate a design effect of 1.5. We have increased the sample size by 20% to allow for loss to follow-up. A total of 17,300 children will need to be screened to recruit 450 children for this trial. The communities are stable and few study children are expected to leave the school during the school year.

This will detect a 20% difference in spectacle wearing, determined using the sampsi command in Stata Statistical Software version 14 (StataCorp, College Station, TX, USA).

### Randomization

A list of schools where the head teachers has given permission will be prepared in India (PM) and each school will be allocated a unique ID. All clusters will be randomized at once so allocation concealment will not be an issue. The schools will be randomized using a web-based randomization service (Sealed Envelope Ltd. 2016. Simple randomisation service [Online]). Available from: https://www.sealedenvelope.com/simple-randomiser/v1/ [Accessed 3 Jan 2017])

The schools will be randomly allocated to intervention or comparator arm, stratified by size, i.e. the number of children enrolled at the school between the age 11–15 years. To reduce contamination, schools will be allocated to the intervention and control arm and not individual children, and study schools will not be closer than 10 km to minimize contact between children in the different arms of the trial. Recruitment bias will be unlikely because all children who fail screening i.e. who have a VA of less than 6/9.5, will have similar procedures thereafter, i.e. refraction, dispensing spectacles or referral, apart from the health education intervention, which will be applied after recruitment. Figure 1 is a flowchart showing a child’s journey and the activities involved from screening, to deciding whether they are eligible for recruitment, then randomization and follow-up.

### Primary outcome

The primary outcome is defined as the proportion of children who are wearing their spectacles at an unannounced visit to the school 3 to 4 months after delivery of the spectacles. A new field worker will be recruited and trained to collect the primary outcome data who will be masked to the hypothesis and intervention arm. Spectacle wear will be ascertained using the four categories defined by Wedner [18] where categories 1 or 2 below define spectacle wearing, and categories 3 or 4 as non-spectacle wearing: (1) wearing the spectacles at the time of the unannounced visit, (2) not wearing the spectacles at the time of the visit but have them at school, (3) not wearing the spectacles at the time of the visit but said they are at home, and (4) not wearing the spectacles at the time of the visit as they are broken or lost.

### Secondary outcome

Uptake of referral to Pushpagiri Eye Institute, which will be assessed 4–5 months after screening.

### Other outcomes

At follow-up, each child not wearing their spectacles will be asked why they are not wearing them. Children wearing spectacles will be asked why they are wearing them. They will be asked if there is a second reason. Their response will be written down verbatim by field workers and coded afterwards, with reasons for non-wear likely to fall into the following categories: (1) never received them, (2) lost, (3) broken or scratched, do not like wearing them because (4) they were teased, or (5) appearance, or (6) headache or eye strain; (7) parents do not like the child to wear them, (8) did not notice an improvement in vision i.e. no benefit and (9) other, which will be specified.

The costs of developing the Peek education package, and for delivering both arms of the trial, will be determined using standard costing methods and data from Peek software developers. The cost per child wearing their spectacles at follow-up will be calculated and compared between arms.

### Data management

All data from both arms of the trial will be entered and stored in the Peek database. Data will be transferred into Stata for analysis.

All field staff will undergo rigorous training in using Peek for screening and entering and recording data. Inter-observer agreement studies will be done for VA screening and refraction.

The Peek Acuity database will be password protected. At the end of the study, the data will be archived at LSHTM.

### Data analyses

Analysis will be in the groups to which children were randomly allocated. We expect all children will be given the spectacles required.

#### Primary outcome

The proportion of children wearing or having their spectacles with them at school at 3 to 4 months will be compared between the intervention and standard arms using the risk difference with 95% confidence intervals adjusted for cluster (school).

A separate analysis will also be undertaken to adjust for factors that may affect spectacle wear such as gender, age (linear term), degree of refractive error in the better seeing eye (linear term), previously wore spectacles (binary data), parental spectacle wear (binary data) and educational level (categorical data) if there are imbalances between the two arms of the trial. There will be no subgroup analysis.

In the Peek software the name of the school will never be entered, only the school study ID and the allocation code. The same applies to schools in the comparator arm. Schools will, therefore, only be identified by ID number while the data are being analysed.

Masked analysis of the primary outcome will be difficult as the database for schools allocated to the intervention arm will a larger number of fields than the comparator arm e.g., the SightSim image the child took home; the number and date of voice messages sent to parents.

#### Secondary outcomes

The proportion of children who access eye care after referral to Pushpagiri Eye Institute will be compared between arms, using the risk difference with 95% confidence intervals adjusted for cluster (school).

#### Other outcomes

Reasons for non-spectacle and spectacle wear will be compared between the two arms of the trial, after categorizing their responses.

Data on the cost per child wearing their spectacles at follow-up will only be analysed should the difference in spectacle wear be 20% or greater in the intervention arm.

### Data monitoring

A data monitoring committee will not be required. There is no reason to expect significant adverse effects. Interim analyses are not planned.

### Harms

Neither arm of the trial has any anticipated harms. If spectacles are prescribed inaccurately, or fitted incorrectly, they can cause blurred vision and/or eyestrain or headaches whilst using the spectacles. In this trial all refractions and spectacle fittings will be undertaken by highly experienced optometrists to ensure inaccurate prescribing is unlikely. When children are followed up at 3–4 months, they will be asked whether these symptoms were the reason they did not wear their spectacles. If a child reports any of these symptoms, they will be refracted again and given new spectacles if required.

### Dissemination

Findings will be reported using CONSORT guidelines for cluster randomized trials. All investigators will contribute to the dissemination strategy, which is likely to include a summary of the findings for the local Steering Committee, head teachers, a report for the website of participating institutions, publications in peer-reviewed journals, presentation at national (UK and India) and international conferences.

### Protocol amendment

Important protocol modifications, such as eligibility criteria, will be reported to the Interventions Research Ethics Committee, LSHTM, the Institutional Ethics Committee at Public Health Foundation of India, the Indian Council of Medical Research and Controlled-Trials.com.

### Consent

Approval for the trial will be sought from the relevant school authorities, including the District Education Officer and by the lead collaborator in India. Written informed consent will be obtained from head teachers, who will be given copies of the information sheet and signed consent forms.

Parents of all children to be screened will be sent an information sheet via the children explaining that their child’s vision will be tested, and they will be given spectacles, if required. Parents will be allowed to opt out. If on the day of screening children whose parents have opted out still want to be screened, the child will be given an assent form to sign which will be countersigned by the head teacher. The child will be given a copy to take home. They will then be screened and given spectacles, if required, but will not be recruited to the trial. The school ID of these children will be entered into the Peek software and they will be given a child ID of 00.

All children recruited to the trial will be given verbal information in the local language about the study and an explanation of the procedures by trained field workers. They will also be given an opportunity to ask questions at the time.

All the information sheets and consent forms will be translated into local languages (Hindi and Telugu).

### Confidentiality

Data will be kept confidential as no identifiers will be entered into the Peek database. Data will be anonymized by allocating a unique study ID for each school and each participant. The Peek database will be password protected. At the end of the study, the data will be archived at LSHTM.

All data will be made readily available in a public domain after the initial analyses and results are published.

### Access to data

Only investigators at LSHTM and the lead investigator at PHFI will have access to the final trial dataset. A memorandum of understanding will be drawn up between the two institutions highlighting intellectual property issues, which will include data sharing and availability of the data at the end of the study.

### Post-trial care

Given that myopia can progress during adolescence it is recommended that school vision screening be repeated every 2 years for this age group. This ensures that children whose spectacles require replacing are identified and children entering the school system for the first time are screened. This process can be put into place with support from Pushpagiri Eye Hospital and the local education authorities.

## Discussion

This trial is designed to evaluate whether a health education package for teachers, parents and children delivered using innovative mobile phone technology (Peek) increases spectacle wear at 3 to 4 months, as well as the uptake of referral of children identified during vision screening with other eye conditions. We will also assess the cost of developing and delivering the health education intervention in the intervention arm of the trial, and the cost of dispensing and delivering the spectacles in both arms of the trial. We will compare the cost per child wearing their spectacles at follow-up in both arms.

The Government of India recognizes the importance of correcting RE in children as they are included in the national programme for child health, called Rashtriya Bal Swasthya Karyakram (RBSK), and the National Program for the Control of Blindness. The health of schoolchildren is also recognized by international health experts, policy makers, governments and international agencies as contributing to child development, learning and socio-economic development. This includes FRESH (Focus Resources on Effective School Health) whose partners include Education International; Partnership for Child Development; UNESCO; UNICEF; the World Food Programme; the World Health Organization and the World Bank. Results of this project will, therefore, be of relevance to FRESH and local, national and international agencies.

### Trial status

At the time of submission, the formative research has been completed and recruitment was ongoing. Recruitment started on 4 January 2017.

## Additional files

Additional file 1: Example of a SightSim image generated by Peek simulating the visual blur caused by uncorrected refractive error. (DOCX 1760 kb)

Additional file 2: SPIRIT checklist. (PDF 36 kb)

## Abbreviations

App: Application
Peek: Portable Eye Examination Kit
RE: Refractive error
uRE: Uncorrected refractive error
VA: Visual acuity
