# Erratum to: Effectiveness of a novel mobile health education intervention (Peek) on spectacle wear among children in India: study protocol for a randomized controlled trial

**DOI:** 10.1186/s13063-017-2071-8

**Published:** 2017-07-07

**Authors:** Priya Morjaria, Andrew Bastawrous, Gudlavalleti Venkata Satyanarayana Murthy, Jennifer Evans, Clare Gilbert

**Affiliations:** 1 Faculty of Infectious and Tropical Diseases, Department of Clinical Research, London School of Hygiene and Tropical Medicine, Keppel Street, London, WC1E 7HT UK; 2 Indian Institute of Public Health, Plot No #1, A.N.V. Arcade, Amar Co-op Society, Kavuri Hills, Madhapur, Hyderabad, 500033 India

## Erratum

In the original publication [1] was the name ‘SightSim’ still used, but this is not correct anymore. The name was frequently used within the article, but should be replaced with the name ‘PeekSim’.

Old name: SightSim

Correct name: PeekSim
