# Supplementary figures and images for: Effectiveness of a novel mobile health education intervention (Peek) on spectacle wear among children in India: study protocol for a randomized controlled trial
Source: Trials. 2017 Apr 8;18:168. doi: 10.1186/s13063-017-1888-5 (PMC5385039; doi:10.1186/s13063-017-1888-5)

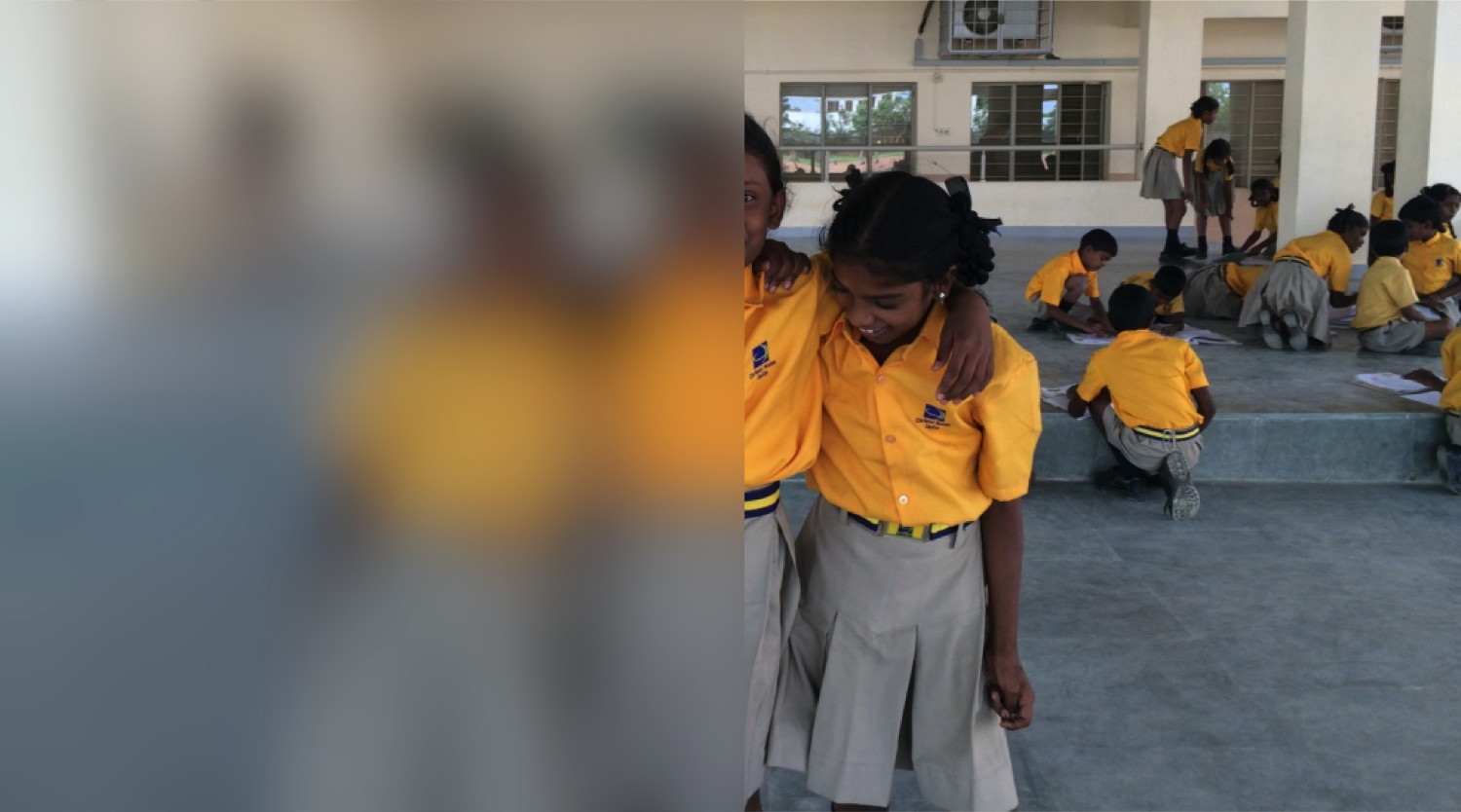

Supplement: Supplementary file 1 — Example of a SightSim image generated by Peek simulating the visual blur caused by uncorrected refractive error. (DOCX 1760 kb) [file 13063_2017_1888_MOESM1_ESM.docx]
